# Catalytic Degradation of Bisphenol A with a Magnetically Recoverable Geopolymer Composite Using Coal Gangue

**DOI:** 10.3390/molecules29153657

**Published:** 2024-08-02

**Authors:** Qishun Shi, Danlei Wu, Chunli Guo, Jianchao Ma

**Affiliations:** 1 College of Materials Science and Engineering, Taiyuan University of Technology, Taiyuan 030024, China; sqshun980904@163.com; 2 College of Mining Engineering, Taiyuan University of Technology, Taiyuan 030024, China; 18635306231@163.com

**Keywords:** coal gangue geopolymer, MnFe_2_O_4_, advanced oxidation processes, Bisphenol A, hydroxyl radical

## Abstract

The widespread presence and use of Bisphenol A (BPA) in aquatic environments has caused significant ecological damage. Coal gangue (CG), a byproduct of coal mining, poses a major environmental concern due to its vast land occupation and potential for pollution. A magnetic recyclable geopolymer (MnFe_2_O_4_-CGP) using coal gangue geopolymer (CGP) as the carrier was successfully synthesized and was evaluated for its ability to Fenton-like degrade BPA. The characterization techniques revealed the successful incorporation of spherical MnFe_2_O_4_ onto the CGP surface and that CGP serves as an excellent platform for the immobilization and dispersion of MnFe_2_O_4_. The degradation rate reached 100% within 60 min at pH = 5, 15 mmol/L H_2_O_2_, 0.6 g/L catalyst, and 50 mg/L BPA, significantly higher than MnFe_2_O_4_ and CGP alone. It was indicated that the degradation rate of BPA in MnFe_2_O_4_-CGP composites was 0.1121 min^−1^, which was consistent with the first-order kinetic model. The saturation magnetization of MnFe_2_O_4_-CGP was measured to be 10.96 emu/g, enabling convenient recovery. MnFe_2_O_4_-CGP exhibited excellent stability, as the degradation rate of BPA remained above 95% even after five reaction cycles. This efficiency may be due to the MnFe_2_O_4_-CGP induced generation of reactive radicals. Quenching and EPR radical trapping experiments unequivocally confirmed that the reactive radical was hydroxyl radical (•OH). These results indicate that MnFe_2_O_4_-CGP has potential application prospects as a magnetic recyclable geopolymer composite in Fenton-like catalysis.

## 1. Introduction

Bisphenol A (BPA), which is a probable endocrine disruptor, is a broadly used phenolic chemical employed in the manufacture of paper, lacquers, plastomers, glues, and epoxies [1]. Its broad application in various industries and consumer products leads to its continued emission into the ecosystem at concerning levels [2,3]. The primary environmental concern regarding BPA is its detrimental impact on bioendocrine systems, even at low nanogram-per-liter concentrations [4]. Consequently, developing effective methods for removing BPA from contaminated water is crucial. Several approaches have been explored for BPA elimination, including wet chemical oxidation [5], physical adsorption [6], biological wastewater treatment [7], and advanced oxidation processes (AOPs) [8].

The Fenton system produces reactive oxygen species (ROS) that non-selectively attack and degrade organic contaminants [9]. Among various metal catalysts, MnFe_2_O_4_ nanoparticles possess a unique spinel structure, offering good chemical stability, soft magnetic properties, and excellent catalytic activity [10]. The synergistic effect between the Mn and Fe redox pairs significantly enhances MnFe_2_O_4_ catalytic activity for H_2_O_2_ activation [11]. Furthermore, MnFe_2_O_4_ nanoparticles are magnetic, enabling their recovery via external magnetic fields [12]. These combined properties make MnFe_2_O_4_ a valuable catalyst in various AOPs.

However, previous studies employing pure MnFe_2_O_4_ nanoparticles as catalysts to degrade methylene blue and tetracycline in Fenton-like systems achieved limited efficiencies (72% and 51.68%, respectively) [13,14]. This may be attributed to the tendency of pure MnFe_2_O_4_ to agglomerate, leading to reduced catalytic activity. Additionally, metal ion leaching from the catalyst can cause secondary pollution, posing a significant environmental challenge [15]. Therefore, identifying a suitable material as a carrier for metal nanoparticles is critical to achieve efficient, easily separable, and green catalysts.

Various materials, such as silica [16], graphene [17], graphitic carbon nitride (g-C_3_N_4_) [18], and TiO_2_ [19], have been used as carriers to enhance the characteristics of MnFe_2_O_4_. Geopolymers offer distinct advantages over these materials: they are stable, cost-effective, environmentally friendly, and readily synthesized [20]. Notably, the unique three-dimensional network structure of geopolymers provides an exceptional platform for immobilizing and dispersing catalytically active components, ultimately enhancing the catalyst activity [21]. Geopolymers, inorganic polymeric materials synthesized from aluminosilicate materials through alkali excitation [22,23], possess robust active sites with formidable reactivity. Their high-density pore structure facilitates the exceptional penetration of desired molecules into the catalyst system [24], rendering geopolymers attractive as catalyst carriers in advanced oxidation processes aimed at degrading organic pollutants [25]. Recently, researchers have explored various geopolymer catalysts for pollutant degradation, such as TiO_2_-based geopolymer microspheres [26], ZnO-based fly ash geopolymers [27], and Cu_2_O-based metakaolin geopolymer composite catalysts [28]. These geopolymer catalysts exhibit satisfactory pollutant degradation effects. Coal gangue (CG), with a high silica and alumina content (approximately 80%), akin to fly ash and slag, emerges as a suitable candidate as a feedstock for geopolymers [29]. CG, a coproduct of the coal extraction and washing processes, despite its wide range of availability, remains underutilized in China [30]. In China, CG causes major environmental problems because of its large quantity and ability to pollute [31]. Reusing coal gangue can mitigate its negative impacts, convert it into resources, and reduce the cost of current carriers or catalysts. Additionally, the use of coal gangue geopolymers as catalyst carriers remains largely unexplored and warrants further research.

In this study, a magnetic recyclable geopolymer (MnFe_2_O_4_-CGP) utilizing CG as the raw material was successfully synthesized and applied to degrade BPA in a Fenton-like reaction. The MnFe_2_O_4_-CGP magnetic composite materials were analyzed using X-ray powder diffraction (XRD), Fourier transform infrared spectroscopy (FT-IR), scanning electron microscopy (SEM), and vibrating sample magnetometry (VSM). The effects of different systems, pH levels, catalyst dosages, and H_2_O_2_ concentrations on the catalytic degradation were discussed. The degradation rate and reaction rate of BPA by MnFe_2_O_4_-CGP were much higher than that by MnFe_2_O_4_ and showed good catalytic stability and a low leaching rate of Fe and Mn ions. Additionally, we will analyze the primary mode of action of the Fenton-like degradation of BPA through complementary quenching experiments.

## 2. Results and Discussion

### 2.1. Characterization of MnFe_2_O_4_-CGP Composite

**Figure 1:**
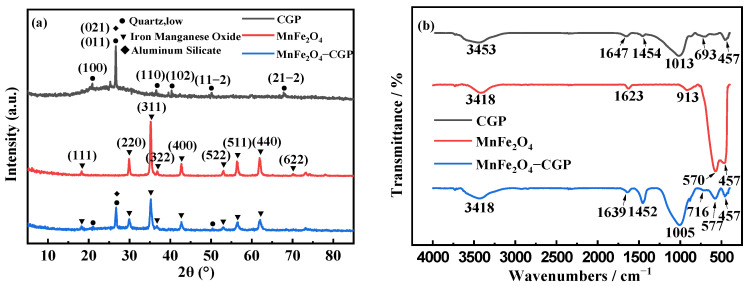
(**a**) XRD patterns and (**b**) FT-IR spectra of CGP, MnFe_2_O_4_, and MnFe_2_O_4_-CGP.

Figure 1a presents the X-ray diffraction (XRD) spectrum of CGP, MnFe_2_O_4_, and MnFe_2_O_4_-CGP. The characteristic peak of CGP is observed in the 2θ range of 17° to 32°. This “bulge peak” signifies the material being in an intermediate state between the amorphous and semicrystalline states, confirming the formation of silica-aluminate hydrate gel in CGP [32]. The dominant peak at 2θ = 26.66° corresponds to the (011) crystal plane of quartz (JCPDF No. 78-1253) and also aligns with the (021) crystal plane of aluminosilicate (JCPDF No. 16-0602). Additionally, at 2θ = 20.9°, 36.56°, 40.34°, 50.12°, and 67.82°, corresponding to the (100), (110), (102), (102), (11-2), and (21-2) crystal planes of quartz (JCPDF No. 78-1253), successful preparation of coal gangue geopolymers is indicated [30]. The most prominent peak of MnFe_2_O_4_ is observed at 2θ = 34.94°, corresponding to the (311) crystal plane, and peaks at 2θ = 18.04°, 29.66°, 36.54°, 42.45°, 52.64°, 56.11°, and 61.59° correspond to the (111), (220), (222), (400), (422), (511), and (440) crystal planes of MnFe_2_O_4_, respectively (JCPDF No. 75-0035), confirming the successful preparation of MnFe_2_O_4_ [10]. The XRD patterns of MnFe_2_O_4_-CGP retained the characteristic diffraction peaks of both CGP and MnFe_2_O_4_, and there was no clear deviation of the peaks at the 2θ value, which showed that the incorporation of MnFe_2_O_4_ would not destroy the structure of CGP. Therefore, the MnFe_2_O_4_-CGP catalyst consisted of CGP and MnFe_2_O_4_. Notably, the “bulge peak” of MnFe_2_O_4_-CGP exhibits a reduced peak area compared to that of CGP. This reduction can be attributed to the introduction of magnetic particles outside the CGP system, leading to a relative reduction in the amorphous gel phase of the geopolymer product [32].

Figure 1b displays the Fourier transform infrared (FT-IR) spectra of CGP, MnFe_2_O_4_, and MnFe_2_O_4_-CGP composites, offering valuable information about their chemical properties. In the spectrum of CGP, the region between 800 cm^−1^ and 1200 cm^−1^ corresponds to the asymmetric telescopic vibration of the Si-O-T bond (where T represents Si or Al), which is a common phenomenon in the formation of geopolymers [30]. Notably, the peaks at 3453 cm^−1^ and 1647 cm^−1^ can be attributed to the -OH tensile vibrations of water molecules and the H-O-H deformation vibrations, respectively, showing the existence of chemically bound water within the material. In the spectrum of MnFe_2_O_4_, the characteristic absorption peaks of the Fe-O and Mn-O bonds appeared at 570 cm^−1^ and 457 cm^−1^, respectively. Additionally, the peaks observed at 3418 cm^−1^ and 1623 cm^−1^ correspond to the tensile vibrations and flexural vibrations of -OH bonding, respectively, suggesting the formation of absorption peaks resulting from hydroxyl groups on the surface of MnFe_2_O_4_ and their involvement in conjoined hydrogen bonding [33]. Finally, the spectrum of MnFe_2_O_4_-CGP reveals important findings. The peak at 1005 cm^−1^ is related to the asymmetric Si-O-T tensile vibrations of CGP. Its absorption peak undergoes a shift towards a lower wavelength number due to the introduction of MnFe_2_O_4_, while also exhibiting an increased peak intensity within the vibrational band. Moreover, the lower wavelength region at 716 cm^−1^ is attributed to the flexion of the SiO_4_/AlO_4_ tetrahedra and symmetric elongation of AlO_4_. Furthermore, the two vibrational bands observed at 577 cm^−1^ and 457 cm^−1^ correspond to the Fe-O tensile vibrations and Mn-O tensile vibrations of MnFe_2_O_4_ [10,34], providing evidence of the successful loading of MnFe_2_O_4_ on the CGP surface.

**Figure 2:**
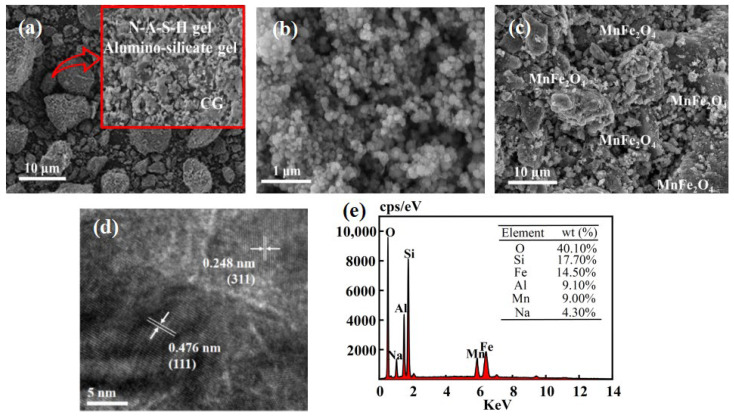
SEM images of (**a**) CGP, (**b**) MnFe_2_O_4_, and (**c**) MnFe_2_O_4_-CGP; (**d**) HRTEM image of MnFe_2_O_4_-CGP and (**e**) EDS of MnFe_2_O_4_-CGP.

Figure 2a displays the scanning electron microscopy (SEM) image of CGP, demonstrating the polymerization occurring on the CGP surface and the dissolution of CGP by the alkali exciter, releasing Si^4+^ and Al^3+^ for polymerization reactions. The CGP is a mixture of NASH gel, silica-aluminate gel, and coal gangue particles [30]. Figure 2b presents the SEM image of MnFe_2_O_4_, revealing the aggregation of nanoparticles with irregular morphology. The reason for that was the strong magnetism and small size of MnFe_2_O_4_. This aggregation hinders the effectiveness of MnFe_2_O_4_ as a catalyst [35]. In contrast, it can be observed from Figure 2c that MnFe_2_O_4_ was homogeneously attached to the surface of CGP, and the agglomeration of MnFe_2_O_4_ was reduced due to the addition of CGP, improving the catalytic activity. Figure 2d illustrates a high-resolution TEM image of a single MnFe_2_O_4_-CGP nanoparticle, where the lattice stripes corresponding to the (311) flat are well visible with a distance of 0.248 nm. Figure 2e shows the EDS pattern of MnFe_2_O_4_-CGP, with peaks corresponding to Si, Al, O, Na, Fe, and Mn, confirming the composition of MnFe_2_O_4_ and demonstrating the successful attachment of MnFe_2_O_4_ to the CGP surface, in line with the results of the XRD analysis.

**Figure 3:**
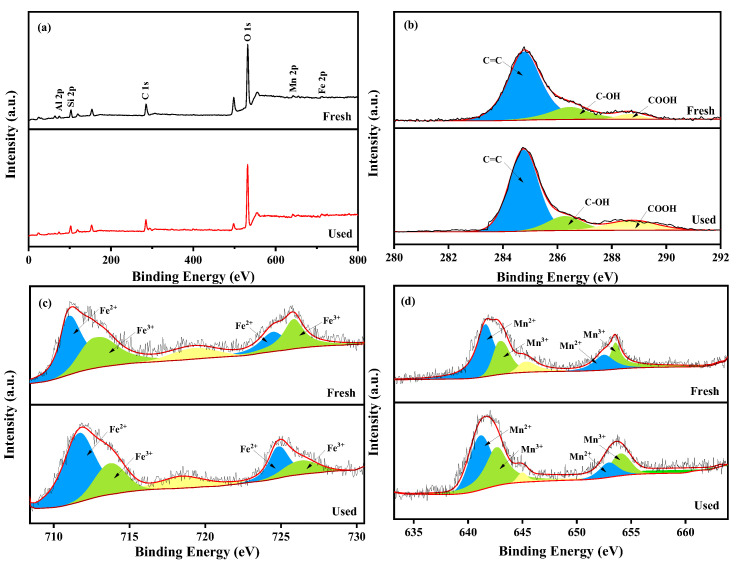
(**a**) XPS survey spectra of MnFe_2_O_4_-CGP composite before and after reaction, (**b**) C 1s, (**c**) Fe 2p, and (**d**) Mn 2p.

As shown in Figure 3, the chemical composition and valence states of fresh and used MnFe_2_O_4_-CGP surface elements were studied by XPS spectral analysis. Elemental peaks of C, O, Fe, and Mn can be observed in the XPS survey spectrum of MnFe_2_O_4_-CGP in Figure 3a. The peaks obtained at 284.9, 531.8, 712.1, and 641.1 eV correspond to C 1s, O 1s, Fe 2p and Mn 2p, respectively. The C 1s spectrum has three peaks at 284.7, 286.3, and 288.8 eV (Figure 3b), corresponding to C=C, C-OH, and HO-C=O, respectively [36,37]. The characteristic peaks of the Fe 2p spectrum have five components as shown in Figure 3c. The peaks at 711.7 and 724.8 eV are from Fe^2+^ in the Fe 2p_3/2_ and Fe 2p_1/2_ orbitals, respectively. Similarly, the peaks at 713.7 and 726.3 eV are from Fe^3+^ in the Fe 2p_3/2_ and Fe 2p_1/2_ orbitals, respectively. The peak at 718.4 eV is the Fe 2p satellite peak [38]. The XPS spectrum of Mn 2p is shown in Figure 3d, with peaks at 642.1 and 653.3 eV belonging to Mn^2+^ in the Mn 2p_3/2_ and Mn 2p_1/2_ orbitals, and peaks at 643.3 and 654.4 eV belonging to Mn^3+^ in the Mn 2p_3/2_ and Mn 2p_1/2_ orbitals [39]. The peak at 645.8 eV is probably attributable to the presence of Mn^2+^.

**Figure 4:**
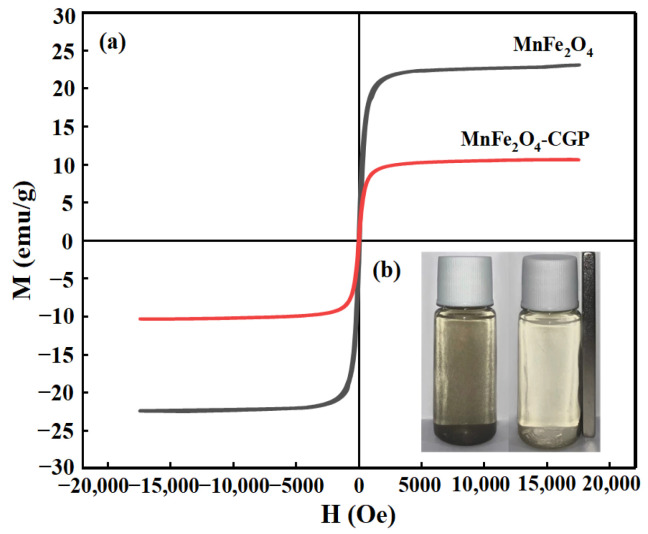
(**a**) Magnetic hysteresis loops for pure MnFe_2_O_4_ and MnFe_2_O_4_-CGP; (**b**) magnetic effect diagram of MnFe_2_O_4_-CGP.

The VSM hysteresis loops of MnFe_2_O_4_ and MnFe_2_O_4_-CGP are illustrated in Figure 4a. The saturation magnetization intensity of MnFe_2_O_4_ nanoparticles is measured at 23.51 emu/g, while MnFe_2_O_4_-CGP exhibits a lower magnetization intensity of 10.96 emu/g. The reduced magnetization of MnFe_2_O_4_-CGP can be due to the existence of non-magnetic CGP carriers. Nevertheless, MnFe_2_O_4_-CGP still retains sufficient magnetic properties for effective separation by an external magnetic field. Figure 4b illustrates that the aqueous solution of the MnFe_2_O_4_-CGP catalyst had good dispersion. When a magnet was placed outside the bottle, the black powder was completely attracted by the magnet within 10 s and the solution became transparent. This indicates that the MnFe_2_O_4_-CGP catalyst has a good magnetic response to the applied magnetic field, which was beneficial for fast magnetic separation and reuse of the aqueous solution. From Figure S1, it is evident that the magnetic recovery rates for cycling five times are 99.3%, 98.5%, 96.1%, 95.3%, and 94.6% under the influence of an external magnetic field. The decline in the recovery rate may be attributed to catalyst degradation during the cycle, with CGP remaining as the residual carrier after magnetic recovery.

### 2.2. Effects on the Degradation of BPA

**Figure 5:**
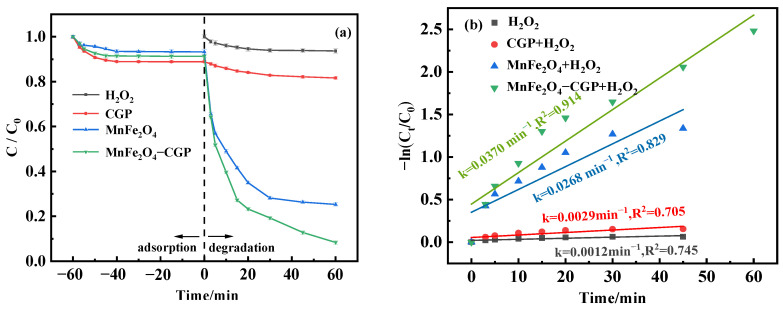
(**a**) Degradation curves and (**b**) kinetic fitting results of BPA with different systems.

The effectiveness of different systems for the degradation of BPA was compared: a single system (H_2_O_2_, and catalyst [CGP, MnFe_2_O_4_, or MnFe_2_O_4_-CGP]), and dual system (catalyst [CGP, MnFe_2_O_4_, or MnFe_2_O_4_-CGP] + H_2_O_2_). To evaluate the effect of different systems on the BPA removal rate, the experimental terms were set: the catalyst was 0.5 mg/L, the pH was 3.5, and the initial BPA was 50 mg/L. As illustrated in Figure 5a, adsorption experiments on BPA were firstly carried out, and the maximum removal of BPA by CGP, MnFe_2_O_4_, and MnFe_2_O_4_-CGP was only 11.13%, 6.71%, and 8.71%, respectively. When 20 mmol/L H_2_O_2_ was added, the removal rate of BPA in the absence of catalyst was only 6.37%. However, when the catalyst was combined with H_2_O_2_, MnFe_2_O_4_ demonstrated a remarkable 74.70% removal of BPA within 60 min, while the MnFe_2_O_4_-CGP composite reached an impressive 91.65% removal efficiency of BPA. These results indicated that catalysis plays a major role in the degradation of BPA rather than adsorption. The catalytic activity of MnFe_2_O_4_-CGP was significantly increased compared to MnFe_2_O_4_. This improvement due to the uniform immobilization of MnFe_2_O_4_ nanoparticles on the CGP surface prevents agglomeration and increases the number of active sites, thus enhancing contact between the BPA molecule and •OH. Consequently, the stability of MnFe_2_O_4_-CGP was enhanced, leading to enhanced Fenton-like efficiency [40]. A first-order kinetic model (Equation (1)) was established through Equation (2) to derive the degradation rate of BPA.

(1) $$\ln\left(\frac{C_{t}}{C_{0}}\right)=\mathrm{kt}$$

where k denotes the kinetic rate constant (min^−1^).

Figure 5b illustrates the correlation between -ln(C_t_/C_0_) and reaction time, suggesting that the degradation of BPA by MnFe_2_O_4_ and MnFe_2_O_4_-CGP was very much in accordance with the first-order model (R^2^ > 0.8). The kinetic rates of H_2_O_2_, CGP + H_2_O_2_, MnFe_2_O_4_ + H_2_O_2_, and MnFe_2_O_4_-CGP + H_2_O_2_ were 0.0012 min^−1^, 0.0029 min^−1^, 0.0268 min^−1^, and 0.0370 min^−1^, respectively. The MnFe_2_O_4_-CGP + H_2_O_2_ system had a higher BPA degradation rate and reaction rate compared to CGP and MnFe_2_O_4_.

**Figure 6:**
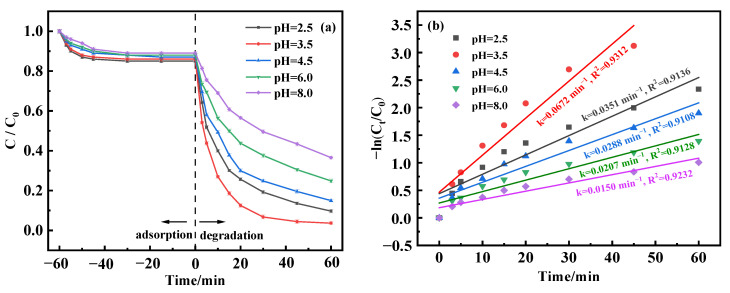
(**a**) Degradation curves and (**b**) kinetic fitting results of BPA at different pH levels.

In Fenton-like processes, the response is markedly influenced by the solution’s pH level [41]. To evaluate how pH variations affect the efficacy of BPA removal, the experimental terms were set: the catalyst was 0.5 mg/L, and the initial BPA was 50 mg/L. The pH of the solution was regulated across a range, with testing at pH values of 2.5, 3.5, 4.5, 6.0, and 8.0 to understand the corresponding effects. Figure 6a illustrates the results; the removal rate of BPA increased with a decrease in pH during the adsorption equilibrium time, reaching a maximum value of 15% at pH = 2.5. When 20 mmol/L H_2_O_2_ was added for Fenton-like catalysis, BPA removal reached 90.34% after 60 min of reaction at pH 2.5. Similarly, at pH 3.5, the BPA removal rate reached 96.34% after 60 min. This can be attributed to the favorable conditions created under an acidic pH, facilitating the accumulation of H_2_O_2_ on MnFe_2_O_4_-CGP and generating •OH species, thus facilitating the conversion of metal ions. The effectiveness of BPA degradation was slightly lessened at a pH of 2.5, possibly attributed to the removal of hydroxyl ions in such a highly acidic environment, as depicted in Equation (2) [42]. Conversely, when the pH was elevated from 2.5 to 3.5, there was a noticeable improvement in the degradation of BPA. This enhancement is credited to the solubilization of the oxide layers on the surface of the MnFe_2_O_4_ within the MnFe_2_O_4_-CGP matrix, boosting the exposure of BPA to novel reactive sites, thereby facilitating the generation of hydroxyl radicals [43]. Nonetheless, with a further pH increase from 3.5 up to 8.0, there was a progressive decline in the degradation efficacy, plummeting from an impressive 96.34% to a lesser 63.48%. This downtrend can be correlated with the hydrolysis of Fe^2+^ and Fe^3+^ at alkaline pH values. These compounds formed by hydrolysis have a diminished efficiency in activating H_2_O_2_ compared to free Fe(II) ions and are more prone to form precipitates that dilute the iron ions available for the reaction [44].

•OH+ e^−^ + H^+^ → H_2_O (2)

From Figure 6b, the degradation rate increased and then decreased with increasing pH level. Initially, the degradation rate was 0.0351 min^−1^ at pH 2.5. The degradation rate decreased from 0.0672 min^−1^ to 0.0150 min^−1^ when the pH was adjusted from 3.5 to 8.0. In addition, the BPA removal at different pH levels fitted well with the first-order model (R^2^ > 0.9). Consequently, the most favorable pH for the degradation of BPA was ascertained to be 3.5.

**Figure 7:**
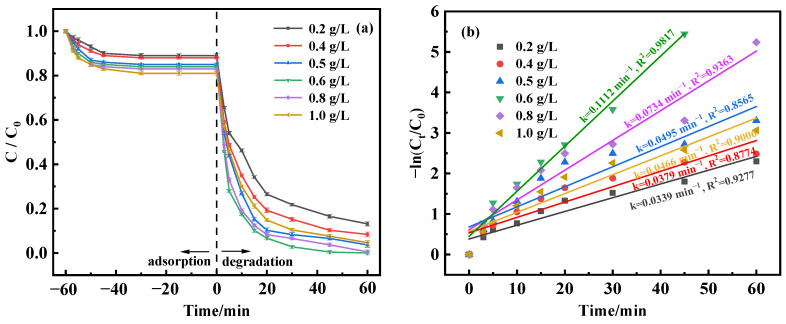
(**a**) Degradation curves and (**b**) kinetic fitting results of BPA at different catalyst dosages.

In an effort to examine how varying quantities of MnFe_2_O_4_-CGP catalyst affect the breakdown of BPA, experimental setups were created to alter the catalyst concentration in the range of 0.2 to 1.0 g/L while maintaining BPA’s initial concentration at 50 mg/L and the pH at 3.5. Figure 7a represents the correlation between different MnFe_2_O_4_-CGP doses and the efficacy of BPA degradation. The results demonstrated that the removal rate of BPA increased with the increase in catalyst dosage during the adsorption equilibrium time, reaching a maximum of 19% at a catalyst dosage of 1.0 g/L. When 20 mmol/L H_2_O_2_ was added for Fenton-like catalysis, there was a positive correlation between the catalyst amount and BPA degradation effectiveness, with BPA being completely degraded at the 0.6 and 0.8 g/L dosages. This observed improvement was attributed to the increased number of active sites on MnFe_2_O_4_-CGP, which boosts •OH radical production and thus accelerates BPA degradation. Nonetheless, surpassing the catalyst dosage of 0.8 g/L led to a decrease in the BPA removal and degradation rates. This decline was presumably attributed to the overabundance of ≡Fe(II) and ≡Mn(II) at the catalyst surface, which potentially consumes •OH radicals, diminishing the BPA removal and degradation rates (Equations (3) and (4)) [34].

≡Fe(II) + •OH → ≡ Fe(III) + OH^−^ (3)

≡Mn(II) + •OH → ≡ Mn(III) + OH^−^ (4)

Figure 7b delineates a trend of increasing and then decreasing degradation rates contingent on the catalyst amount, with the highest degradation rate at 0.1112 min^−1^ with a 0.6 g/L dosage. In addition, the removal of BPA at different catalyst doses fitted well with the first-order model (R^2^ > 0.8). Therefore, the ideal catalyst dosage for BPA’s optimal degradation is identified to be 0.6 g/L.

**Figure 8:**
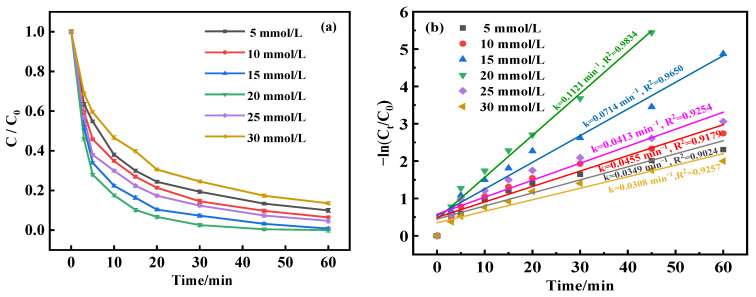
(**a**) Degradation curves and (**b**) kinetic fitting results of BPA at different H_2_O_2_ concentrations.

In the quest to determine the impact of the H_2_O_2_ concentration on BPA degradation in the MnFe_2_O_4_-CGP catalyst system, an experimental study was conducted. As depicted in Figure 8a, the H_2_O_2_ dosages were varied from 5 to 30 mmol/L under constant conditions: BPA initial concentration of 50 mg/L, catalyst concentration of 0.6 mg/L, and pH stabilized at 5. The results showed a progressive increase in the BPA degradation rates with a rise in H_2_O_2_ concentration from 5 up to 20 mmol/L, with degradation reaching 100% at both 15 and 20 mmol/L. This can be interpreted as the enhanced generation of •OH from the decomposition of H_2_O_2_, which actively facilitates the breakdown of BPA. Conversely, when H_2_O_2_ levels were increased to between 25 and 30 mmol/L, BPA degradation dropped noticeably to 86.46%. This decrease could be due to an excessive presence of H_2_O_2_ leading to a reaction of the •OH to form HO_2_•, which possesses a very low oxidation potential in the beginning stages of the reaction, as demonstrated in Equations (5) and (6). Meanwhile, an overabundance of •OH radicals may diminish their interactions with organic substances, favoring their recombination (as proposed in Equation (7)), which decreases the rate of BPA degradation [45].

H_2_O_2_ + •OH → H_2_O + HO_2_• (5)

HO_2_• + •OH → H_2_O + O_2_ (6)

2•OH + 2•OH → 2 H_2_O + O_2_ (7)

The degradation kinetics, as observed in Figure 8b, revealed first an increase and then a decrease in the degradation rate corresponding with rising H_2_O_2_ levels, with the highest degradation rate of 0.1121 min^−1^ at a H_2_O_2_ dosage of 15 mmol/L. In addition, the removal of BPA at different catalyst doses fitted well with the first-order model (R^2^ > 0.9). Therefore, the ideal dosage of H_2_O_2_ for the most effective degradation of BPA was determined to be 15 mmol/L.

In order to further evaluate this material, the degradation effect of bisphenol A was compared with that of other catalysts reported in recent years [46,47,48,49,50,51]. The results are shown in Table S1. Compared with other catalysts, MnFe_2_O_4_-CGP showed relatively high BPA degradation performance in a short period of time at a higher initial concentration of BPA, which has practical application potential.

### 2.3. Adsorption Kinetics and Isotherms

Since the removal of BPA included both adsorption and Fenton-like oxidation, it is necessary to analyze the adsorption kinetics and isotherms. In this study, the adsorption mode of MnFe_2_O_4_-CGP on BPA was investigated using the pseudo-first-order kinetic model and the pseudo-second-order kinetic model, which were formulated by Equations (8) and (9), respectively.

(8) $$\ln\left(q_{e}-q_{t}\right)=\ln q_{e}-k_{1}t$$

(9) $$\frac{t}{q_{t}}=\frac{1}{k_{2}q_{e}^{2}}+\frac{t}{q_{e}}$$

where q_t_ (mg/g) and q_e_ (mg/g) are the adsorption amounts of BPA at time t and equilibrium, respectively, and k_1_ (min^−1^) and k_2_ (g·mg^−1^·min^−1^) are the rate constants of the pseudo-first-order and pseudo-second-order kinetic models, respectively.

The pseudo-first-order and pseudo-second-order kinetic models for the MnFe_2_O_4_-CGP adsorbing of BPA are shown in Figure S2, and the corresponding kinetic parameters are detailed in Table 1. Two kinetic simulation results were compared, and it was discovered that the correlation coefficient fitting R^2^ (0.997) of the pseudo-second-order kinetic model was significantly better than that of the pseudo-first-order kinetic model (0.993). This indicated that the adsorption of BPA by MnFe_2_O_4_-CGP could be described with a pseudo-second-order kinetic model, which further confirmed that the adsorption process belonged to chemisorption [52].

The Langmuir model and Freundlich model are the two most commonly used adsorption isothermal models, which were formulated by Equations (10) and (11), respectively.

(10) $$\frac{C_{e}}{q_{e}}=\frac{1}{q_{m}K_{L}}+\frac{C_{e}}{q_{m}}$$

(11) $$\ln q_{e}=\ln K_{F}+\frac{1}{n}\ln C_{e}$$

where q_m_ (mg/g) is the maximum adsorption capacity of MnFe_2_O_4_-CGP at a certain temperature, C_e_ (mg/L) is the equilibrium concentration, K_L_ (L/mg) is the Langmuir constant related to the adsorption energy, K_F_ is the Freundlich adsorption isotherm constant related to the adsorption degree, and n is related to the adsorption strength.

Figure S3 illustrates the Langmuir and Freundlich isotherm models for the MnFe_2_O_4_-CGP composite material, with the corresponding isotherm parameters detailed in Table 2. It can be revealed by comparing Figure S3 and Table 2 that the Langmuir model of MnFe_2_O_4_-CGP has an R^2^ of 0.965, which is higher than that of the Freundlich model of 0.686. Therefore, the adsorption process of MnFe_2_O_4_-CGP composites on BPA was more composite Langmuir model. This result indicated that the process is a monolayer adsorption. The maximum adsorption of BPA by MnFe_2_O_4_-CGP was calculated as 14.84 mg/g by the Langmuir isotherm model.

### 2.4. Stability and Reusability

**Figure 9:**
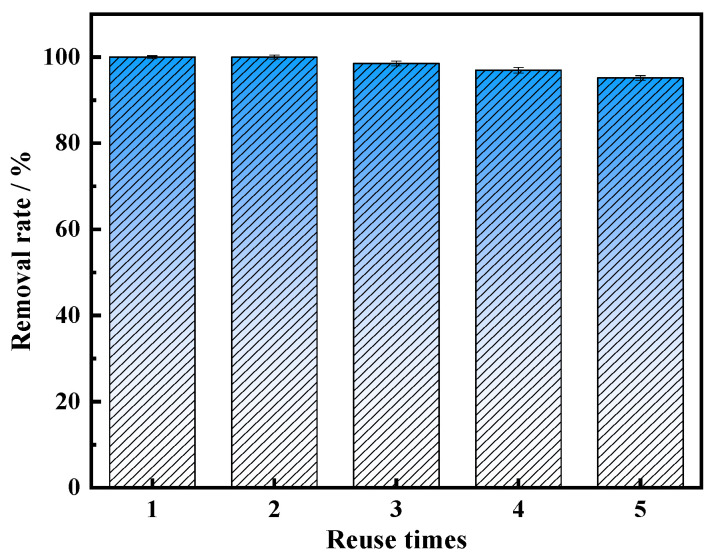
The reusability of MnFe_2_O_4_-CGP catalyst for BPA degradation (H_2_O_2_ = 15 mmol/L, MnFe_2_O_4_-CGP = 0.5 g/L, [BPA]_0_ = 50 mg/L, and pH = 2.5).

In the actual phenol-containing wastewater treatment process, the reproducible performance of the catalyst is essential. In order to assess the stability of MnFe_2_O_4_-CGP composites, five consecutive catalytic experiments were conducted under optimal conditions, with the findings displayed in Figure 9. The data showed that BPA was completely eliminated in the first two uses. When used in the third use, the removal rate of BPA was 98.52%, which was not significantly different compared with the first use. This minor decline might be due to the accumulation of BPA on the catalyst’s surface after repeated usage, resulting in a reduction of the catalyst reaction active site. In the fourth and fifth cycles, the BPA removal efficiency dropped to 96.97% and 95.16%, respectively, which were 3.03% and 4.84% lower compared to the first use. The slight decrease could possibly be attributed to a loss of reactive sites on the catalyst and the dissolution of some metal ions from the MnFe_2_O_4_ on the CGP surface, which could cause the material to become less active. In general, the removal rate of BPA can still reach more than 95% after five consecutive uses, the catalytic performance is still good, and the material still has good magnetic properties after use. Iron ion dissolution experiments revealed that the total iron dissolved in the leachate was 1.204 mg/L, with Fe^3+^ accounting for 0.832 mg/L and Fe^2+^ for 0.372 mg/L, which can be considered negligible compared to the dosage of MnFe_2_O_4_-CGP. This indicates the relative stability of MnFe_2_O_4_-CGP.

### 2.5. Identification of Reactive Radical Species

**Figure 10:**
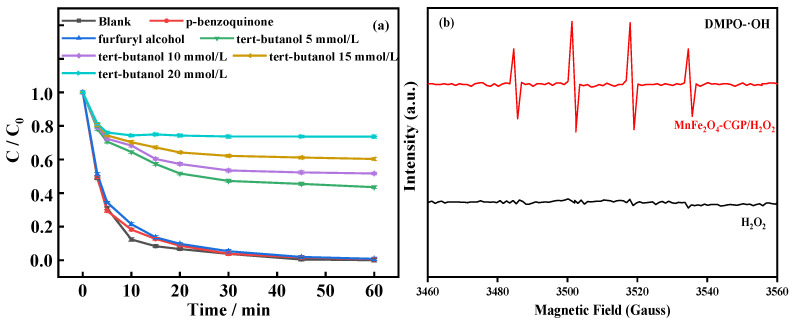
(**a**) Quenching effect of different quenching agents; (**b**) EPR spectra in different systems.

To discern the primary reactive species generated in the MnFe_2_O_4_-CGP/H_2_O_2_ system, 15 mmol/L tert-butanol [53], p-benzoquinone [54], and furfuryl alcohol [55] were selected as quenching agents for quenching experiments with •OH, •O_2_^−^, and ^1^O_2_ respectively (H_2_O_2_ = 15 mmol/L, MnFe_2_O_4_-CGP = 0.6 g/L, [BPA]_0_ = 50 mg/L, and pH = 3.5). The results are presented in Figure 10a. Without the quenching agent, BPA was entirely degraded by MnFe_2_O_4_-CGP. However, the introduction of p-benzoquinone and furfuryl alcohol resulted in negligible changes in BPA degradation, registering 99.39% and 99.17%, respectively. On the other hand, with tert-butanol added to the system, a substantial reduction in BPA degradation was observed, plummeting to 39.69%. In the Fenton system, •OH is typically the reactive oxygen species generated. The impact of different concentrations of tert-butanol on the BPA degradation efficiency was further examined. At a concentration of 5 mmol/L, there was a notable inhibition, with the BPA removal rate dropping to 56.50% from complete degradation. Increasing the tert-butanol to 10 mmol/L led to a further reduction in the removal rate to 48.35%, while at 20 mmol/L, it dropped to 26.35% in the MnFe_2_O_4_-CGP/H_2_O_2_ system. The removal rate of tert-butanol supplemented with 20 mmol/L was decreased from 100% to 26.35%. These declines are indicative of the interaction between tert-butanol and •OH radicals, resulting in the formation of highly selective or inert intermediates. As a potent inhibitor of •OH, tert-butanol effectively suppresses the •OH oxidation reaction. The inhibitory effect gradually intensifies with increasing tert-butanol concentration in the solution.

In order to further determine the types of free radicals generated by the MnFe_2_O_4_-CGP Fenton-like catalytic process, EPR analyses were carried out with the spin trapping agent 5,5-dimethyl-1-pyrroline-N-oxide (DMPO) for the H_2_O_2_ and MnFe_2_O_4_-CGP/H_2_O_2_ systems, and the results are shown in Figure 10b. It was found that a typical DMPO-•OH signal appeared in the MnFe_2_O_4_-CGP/H_2_O_2_ system, which directly proved the presence of •OH. However, the DMPO-•OH signal did not appear in the single H_2_O_2_ system. These findings underscore the significant role of •OH radicals in the MnFe_2_O_4_-CGP/H_2_O_2_ Fenton-like system, which drives the oxidative degradation of BPA.

### 2.6. Possible Catalytic Mechanism

**Figure 11:**
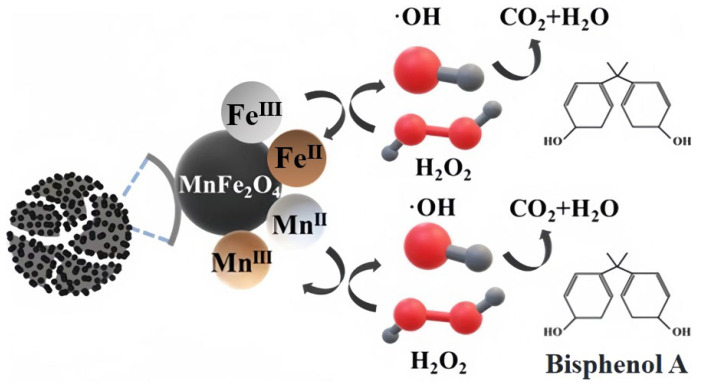
MnFe_2_O_4_-CGP Fenton-like catalytic reaction mechanism.

Based on the above studies, the catalyst mechanism of MnFe_2_O_4_-CGP composites was investigated. The MnFe_2_O_4_-CGP catalyst can activate H_2_O_2_ to produce •OH radicals, which can degrade BPA by breaking the organic chain of BPA. To investigate the active species generated in the active site, fresh and used MnFe_2_O_4_-CGP catalysts were analyzed using XPS, and the results are shown in Figure 3. The peaks of C 1s, O 1s, Fe 2p, and Mn 2p can be observed from Figure 3a, and the surface substances of the catalyst were basically unchanged after 60 min of catalytic reaction, which further confirmed the stability of the MnFe_2_O_4_-CGP catalyst. In the Fenton-like system, the interaction between Fe and Mn can produce a synergistic effect, which drives the generation of free radicals for the degradation of pollutants. Before and after the catalytic reaction, the relative content of Fe^2+^ increased from 29.98% to 43.34%, and the relative content of Fe^3+^ decreased from 70.02% to 56.66% (Figure 3c). Similarly, the percentage of Mn^2+^ decreased from 66.36% to 55.27%, and the percentage of Mn^3+^ increased from 33.64% to 44.73% (Figure 3d). These changes indicated that Fe^2+^ and Mn^3+^ in the MnFe_2_O_4_-CGP catalyst were converted to Fe^3+^ and Mn^2+^ during the Fenton-like reaction and that both the Fe^2+^/Fe^3+^ and Mn^2+^/Mn^3+^ redox pairs were involved in the activation of H_2_O_2_ by the MnFe_2_O_4_-CGP, which led to the generation of •OH radicals. This is a key factor in the removal of BPA by MnFe_2_O_4_-CGP. Based on the above analysis, the mechanism of the MnFe_2_O_4_-CGP Fenton-like catalytic reaction is shown in Figure 11.

## 3. Materials and Methods

### 3.1. Materials

Coal gangue (CG) was obtained from Datong Coal Mine (Datong, China). Bisphenol A (C_6_H_6_O) was purchased from Tianjin Damao Chemical Reagent Factory (Tianjin, China). Sodium hydroxide (NaOH), concentrated hydrochloric acid (HCl), hydrogen peroxide (H_2_O_2_, wt.% 30%), tert-butanol (C_4_H_10_O), anhydrous ethanol (C_2_H_6_O), and methanol (CH_3_OH) were obtained from Tianjin Comio Chemical Reagent Development Center (Tianjin, China). Ferric chloride hexahydrate (FeCl_3_·6H_2_O) and manganese chloride tetrahydrate (MnCl_2_·4H_2_O) were purchased from Tianjin BASF Chemical Co., Ltd. (Tianjin, China). Sodium silicate (Na_2_SiO_3_) was purchased from Shandong Urso Chemical Technology Co., Ltd. (Heze, China) with a modulus of 3.3 and a chemical composition (wt.%) of Na_2_O = 8.3, SiO_2_ = 26.5, and H_2_O = 65.2. All chemicals were analytically pure, except for methanol, which was chromatographically pure.

### 3.2. Characterization

The physical phase constituents of materials were analyzed using the DX-2700 X-ray photoelectron spectrometer (XRD) from China with a scanning speed of 4°/min and a range of 2θ = 5–85°. The presence of functional groups and the character of chemical bonds in the material were analyzed using a Tensor 27 Fourier-transform infrared (FT-IR) spectrometer from Bruker, Germany, with a wavelength of 4000–400 cm^−1^. The morphology and elemental constitution of the material were obtained using a JSM-7800F scanning electron microscope (SEM) from JEOL, Tokyo, Japan, and an X-Max N energy-dispersive spectrometer (EDS) from Tescan, Czechia. The nanostructure of the material was determined using an H-7650 high-resolution transmission electron microscope (HRTEM) from Hitachi, Tokyo, Japan. The magnetic property of the material was analyzed using the VSM-220 vibrating sample magnetometer (VSM) from Changchun, China.

### 3.3. Synthesis of MnFe_2_O_4_ Nanoparticles

A solution was produced by stirring 5.00 g of NaOH with 100 mL of deionized water in a water bath. Separately, 2.00 g of MnCl_2_-4H_2_O and 4.00 g of FeCl_3_-6H_2_O were dissolved in 50 mL of deionized water, resulting in an orange–yellow mixture. The orange–yellow mixture was dropped onto the NaOH solution while keeping the water bath temperature at 95 °C. After the drops were added, the mixture was aged at 95 °C for 2 h. Subsequently, the resulting black–brown material was separated using an external magnet, washed, and desiccated at 80 °C for 12 h. The desiccated MnFe_2_O_4_ was then removed and subjected to grinding.

### 3.4. Synthesis of MnFe_2_O_4_-CGP Composite

**Figure 12:**
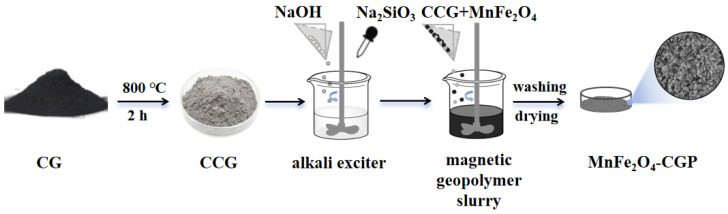
Preparation process of MnFe_2_O_4_-CGP.

CG (ground and sieved to 200 mesh) was subjected to heat activation by cooling it to indoor temperature in a muffle furnace of 800 °C for 2 h. Activated coal gangue (CCG) was then used for subsequent steps, and the molecular composition results of the CCGs are shown in Table 3. The mixture of 1.50 g of MnFe_2_O_4_ and 0.88 g of CCG was combined with 0.19 g of NaOH and 1.00 g of Na_2_SiO_3_ base activator in a plastic beaker. The mixture underwent a reaction at indoor temperature for 30 min. The resulting sample was desiccated at 80 °C for 24 h, washed to neutral pH after drying, and subsequently dried and ground to a particle size of 200 mesh to obtain MnFe_2_O_4_-CGP. The preparation process of MnFe_2_O_4_-CGP was carried out according to the steps shown in Figure 12.

### 3.5. Fenton-like Oxidation

A 150 mL beaker was filled with 100 mL of BPA solution at 50 mg/L concentration. To adjust the pH of this solution, 0.1 mol/L HCl and NaOH were added. The MnFe_2_O_4_-CGP material was introduced into the solution, and after 60 min of adsorption to achieve adsorption balance, H_2_O_2_ was introduced. The resulting sample for analysis was obtained by separating MnFe_2_O_4_-CGP using a magnet and passing it through a 0.22 μm membrane filter at various time intervals, including 3, 5, 10, 15, 20, 30, 45, and 60 min.

To determine the residual BPA concentration by high-performance liquid chromatography (HPLC) using a ZORBAX SB-C18 column, the chromatographic mobile phase consisted of a mixture of methanol and water at a ratio of 70:30. The BPA removal rate, denoted as η, was calculated using the following Formula (12):

(12) $$\eta \left(\%\right)=\frac{C_{0}-C_{t}}{C_{0}}\times 100$$

where C_0_ is the initial concentration of BPA in solution and C_t_ is the concentration of BPA in solution after t min of catalytic reaction. The experimental data were averaged after three experiments to ensure a minimum relative error (5%).

## 4. Conclusions

A magnetic recyclable geopolymer (MnFe_2_O_4_-CGP) was successfully prepared. The study also characterized the MnFe_2_O_4_-CGP using various analytical techniques, such as XRD, FTIR, and SEM. These analytical techniques indicate that MnFe_2_O_4_ was successfully loaded onto CGP and that CGP serves as an excellent platform for the immobilization and dispersion of MnFe_2_O_4_. The MnFe_2_O_4_-CGP catalyst exhibited a BPA removal rate of approximately 100% within 60 min, and its performance was significantly better than that of the single MnFe_2_O_4_. Beyond its impressive catalytic capabilities, MnFe_2_O_4_-CGP also demonstrated notable durability and the ability to be used multiple times. Its magnetic properties were confirmed by VSM tests, which showed that MnFe_2_O_4_-CGP has a magnetic saturation strength of 10.96 emu/g. The catalytic performance of MnFe_2_O_4_-CGP remained high even after five consecutive uses, with BPA degradation rate of 95.16%. These results indicate the favorable magnetic properties and stability of MnFe_2_O_4_-CGP. This study showed that the degradation of BPA by MnFe_2_O_4_-CGP conformed to a first-order kinetic model, and the reaction rate was 0.1121 min^−1^. Tert-butanol quenching and radical trapping experiments emphasized the crucial role of •OH radicals in the catalytic degradation of phenol by MnFe_2_O_4_-CGP. Therefore, this study provides a theoretical basis for the development of Fenton-like catalysts for the degradation of organic pollutants, which can be explored in the future for practical applications in the treatment of phenol-containing wastewaters in industry and catalyst recovery methods.

## Figures and Tables

**Table 1:** Kinetic parameters of pseudo-first-order and pseudo-second-order for the adsorption of BPA.

Pollutant	C_0_ (mg/L)	q_e_ (mg/g)	Pseudo-First-Order Model			Pseudo-Second-Order Model		
			q_1e_ (mg/g)	k_1_ (min^−1^)	R_1_^2^	Q_2e_ (mg/g)	k_2_ (g·mg^−1^·min^−1^)	R_2_^2^
BPA	50	11.667	10.980	0.1779	0.993	12.195	0.0376	0.997

**Table 2:** Adsorption equilibrium isotherm models of MnFe_2_O_4_-CGP.

	Langmuir			Freundlich		
	q_m_ (mg/g)	k_L_ (L/mg)	R^2^	n	k_F_	R^2^
MnFe_2_O_4_-CGP	14.84	0.0690	0.965	2.80	2.8588	0.686

**Table 3:** CCG chemical composition and content.

Component	SiO_2_	Al_2_O_3_	CaO	TiO_2_	Fe_2_O_3_	P_2_O_5_	Other
Content (wt.%)	55.30	42.68	0.18	0.48	0.41	0.46	0.49

## Data Availability

The original contributions presented in the study are included in the article (and Supplementary Material), further inquiries can be directed to the corresponding authors.

## Supplementary Materials

The following supporting information can be downloaded at: https://www.mdpi.com/article/10.3390/molecules29153657/s1, Figure S1. External magnetic field recovery of MnFe_2_O_4_-CGP; Figure S2. (a) Pseudo-first-order dynamics model and (b) pseudo-second-order dynamics model; Figure S3. (a) Fitting of adsorption isotherms for linear MnFe_2_O_4_-CGP Langmuir model and (b) fitting of adsorption isotherms for linear MnFe_2_O_4_-CGP Freundlich model; Table S1 Comparison with other catalysts for the degradation of BPA.
