# Supplementary material for: Catalytic Degradation of Bisphenol A with a Magnetically Recoverable Geopolymer Composite Using Coal Gangue
Source: Molecules. 2024 Aug 2;29(15):3657. doi: 10.3390/molecules29153657 (PMC11313870; doi:10.3390/molecules29153657)
Supplement: Supplementary file 1 [file molecules-29-03657-s001.zip › molecules-3028421-supplementary.pdf]

Supporting Information

For

Catalytic Degradation of Bisphenol A with a Magnetically Recoverable Geopolymer Composite Using Coal Gangue

Qishun Shi [1], Danlei Wu [2], Chunli Guo [1,*] and Jianchao Ma [2,*]

[1] College of Materials Science and Engineering, Taiyuan University of Technology, Taiyuan 030024, China; 740150473@qq.com
[2] College of Mining Engineering, Taiyuan University of Technology, Taiyuan 030024, China; 390379381@qq.com
* Correspondence: guochunli@tyut.edu.cn (C.G.); majianchao@tyut.edu.cn (J.M.)

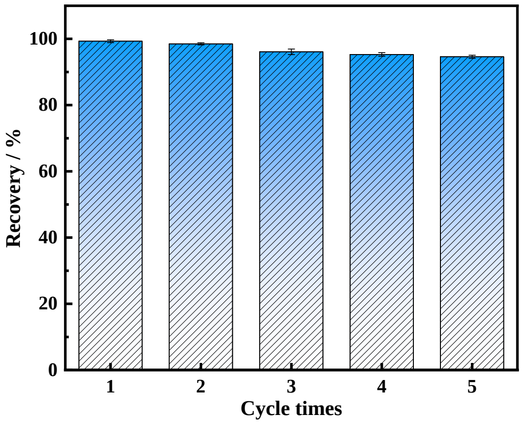

Figure S1. External magnetic field recovery of $MnFe_2O_4$-CGP

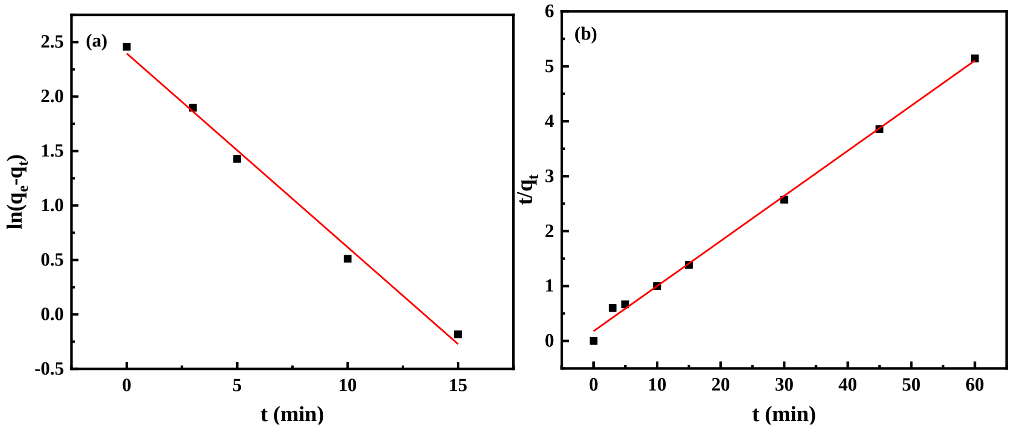

Figure S2. (a) Pseudo-first-order dynamics model; (b) Pseudo-second-order dynamics model

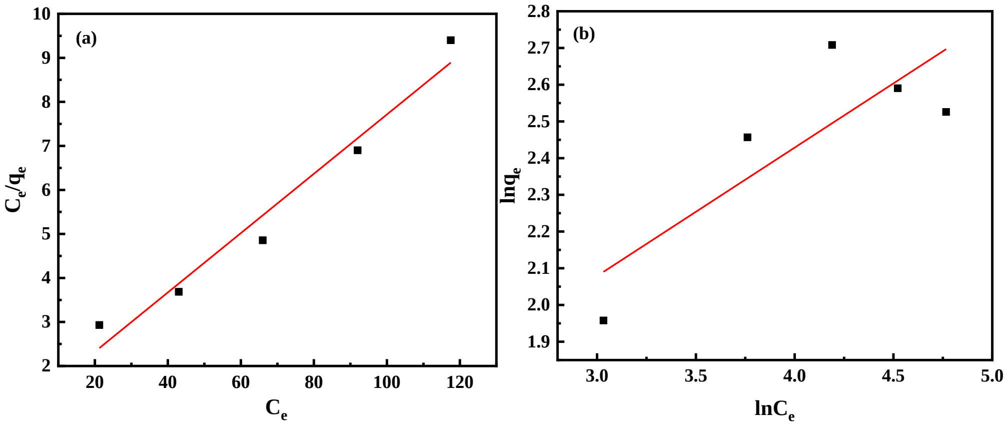

Figure S3. (a) Fitting of adsorption isotherms for linear $MnFe_2O_4$-CGP Langmuir model; (b) Fitting of adsorption isotherms for linear $MnFe_2O_4$-CGP Freundlich model.

Table S1 Comparison with other catalysts for the degradation of BPA

Catalyst	Initial concentration of BPA	Reaction conditions	Efficiency	Reference
$MnFe_2O_4$-CGP	50 mg·L^{-1}	pH = 3.5, H_2O_2 = 15 mmol·L^{-1}, catalyst = 0.6 g·L^{-1}	100%, 60 min	This work
$Ni_xCo_{3-x}O_4$(NCO-2)	10 mg·L^{-1}	pH = 7, catalyst = 0.3 g·L^{-1}, PMS = 2.5 g·L^{-1}	60.3%, 30 min	47
GS-Fe-NPs	25 mg·L^{-1}	pH = 6.9, H_2O_2 = 1.0 mol·L^{-1}, catalyst = 0.3 g·L^{-1}	96.4%, 150 min	48
Fe/N co-doped biochar	10 mg·L^{-1}	pH=6.76, catalyst = 0.1 g·L^{-1}, PMS = 0.5 mmol·L^{-1}	97%, 60 min	49
MnO/MnO-CuMnO (MMCM)	10 mg·L^{-1}	catalyst = 0.2 g·L^{-1}, PDS = 2 mmol·L^{-1}	100%, 120 min	50
$Mn_{0.27}FeO_{4.55}$	10 mg·L^{-1}	pH=7.0, catalyst = 0.5 g·L^{-1}, PMS = 2 mmol·L^{-1}	97.8%, 90 min	51
a composite of graphitized carbon and nZVI (GC-nZVI)	25 μmol·L^{-1}	pH=6.0, H_2O_2 = 0.5 mmol·L^{-1}, catalyst = 0.05 g·L^{-1} UVA irradiation	89%, 60 min	52

References (the serial number of the references corresponds to the order in the main article)

47. Wu, Z.B.; Liang, Y.S.; Zou, D.S.; Yuan, X.Z.; Xiao, Z.H.; Deng, Y.C.; Zhou, Y.Y.; Jiang, L.B.; Qin, P.F. Enhanced heterogeneous activation of persulfate by $NixCo_{3-X}O_4$ for oxidative degradation of tetracycline and bisphenol A. *J. Environ. Chem. Eng.* **2020**, *8*, 104451. doi:10.1016/j.jece.2020.104451.
48. Guo, B.; Xu, T.T.; Zhang, L.; Li, S. A heterogeneous Fenton-like system with green iron nanoparticles for the removal of bisphenol A: Performance, kinetics and transformation mechanism. *J. Environ. Manage.* **2020**, *272*, 111047. doi:10.1016/j.jenvman.2020.111047.
49. Xu, L.; Fu, B.R.; Sun, Y.; Jin, P.K.; Bai, X.; Jin, X.; Shi, X.; Wang, Y.; Nie, S. Degradation of organic pollutants by Fe/N co-doped biochar via peroxymonosulfate activation: Synthesis, performance, mechanism and its potential for practical application. *Chem. Eng. J.* **2020**, *400*, 125870. doi:10.1016/j.cej.2020.125870.
50. Zhu, Y.H.; Sun, Z.J.; Deng, Y.; Liu, F.D.; Ruan, W.Q.; Xie, L.J. Mn_2O_3/Mn_3O_4-$Cu_{1.5}Mn_{1.5}O_4$ Spinel as an efficient Fenton-like catalyst activating persulfate for the degradation of bisphenol A: Superoxide radicals dominate the reaction. *Sci. Total Environ.* **2022**, *839*, 156075. doi:10.1016/j.scitotenv.2022.156075.

51. Ma, Y.H.; Wang, D.L.; Xu, Y.; Lin, H.; Zhang, H. Nonradical electron transfer-based peroxydisulfate activation by a Mn−Fe bimetallic oxide derived from spent alkaline battery for the oxidation of bisphenol A. *Journal of Hazardous Materials* **2022**, *436*, 129172. doi:10.1016/j.jhazmat.2022.129172.
52. Cai, M.J.; Li, J.J.; Wu, F.; Voyard, G.; Mailhot, G.; Brigante, M. Synergistic degradation of bisphenol A in heterogeneous Fenton and photo-Fenton systems catalyzed by graphitized carbon-nano zero valent iron. *J. Environ. Chem. Eng.* **2023**, *11*, 110959. doi:10.1016/j.jece.2023.110959.
